# Transactions of the American Gynecological Society

**Published:** 1878-09

**Authors:** 


*Transactions of the American Gynecological Society.* Vol. II. For the Year 1877. Boston: Houghton, Osgood & Co. Buffalo: Peter Paul & Bro. Octavo, pp. 697. Price, $6.50.

This volume reflects much credit upon the Gynecological Society. Its well filled interior and its fine appearance are very attractive and markedly indicative of ability, industry and resources. Numerous interesting papers by members are followed by valuable discussions by leading men of our country in the specialty, on the subjects presented and thus a breadth of view and wisdom of conclusion are obtainable therefrom that are not to be gathered from the works of single authors. Besides this, by the meeting of distinguished authorities and the earnest labor of discussion, opinions are well sifted and the limits of knowledge better defined than could be without association work. Labor in the directions which are of present interest is organized, aid and facilities are interchanged, the time of reaching satisfactory conclusions greatly expedited, thoroughness of investigation secured, the number of the subjects unfolded, increased and general progress of science in various departments is greatly furthered by the association of investigators. We may fairly expect that in this way much of that which has been mysterious or ill-defined, in the field of gynecology will be cleared up.

The meeting of the Society was held last year in Boston, this year it will be held at Philadelphia on the eleventh, twelfth and thirteenth of September. Its membership at present includes forty-six fellows beside those who have been elected to honorary fellowship. It is the purpose of the Society to have the volume of the transactions of such value that it shall have extended sale.

The Address of the President, Fordyce Barker, M. D., is straightforward and earnest, advocating the maintenance of activity by the members of the Society and of whole souled devotion to truth-seeking in the department. John C. Dalton, M. D., by request, prepared a paper on the Corpus Luteum, true and false, which is given in the volume and accompanied by twelve fine chromo-lithographic plates, representing sections made in ovaries of menstruations and of pregnancy. Thirty-two sets of specimens of ovaries, with histories, were obtained by him as a basis for the paper and these are all described and such pictures of these as are desirable, accompany the report. The most lengthy discussion is that which follows the paper of Ely Van de Warker, M. D., of Syracuse, N. Y., on "The Present States of the Intra-Uterine Stem in the treatment of Flexions of the Uterus. The writer quotes history and authorities in favor of the use of the instrument and advocates its employment when desirable. Drs. Chadwick, Peaslee, Thomas, Goodell, A. H. Smith, W. L Atlee, Wilson of Baltimore, Byford, Skene, Garrigues and J. B. S. Jackson, participate in the discussion of the paper during which the subject of pessaries is considered at length. The distinguished character of the speakers and the opposing views given, make the discussions of interest to readers. Vaginal Ovariotomy, by William Goodell, M. D ; Battey's Operation, by Robert Battey, M. D.; Amputation and Excision of the Cervix Uteri, by John Byrne, M. D.; Sarcoma of the Ovaries, by W. L. Atlee, M. D.; The value of Electrolysis in Ovarian Tumors, by Paul F. Mundé, M. D ; Absence and Atresia of the Vagina, by Thomas N. Emmet, M. D.; Important Points connected with Ovariotomy, by Gilman Kimball, M. D ; Rapid Dilatation of the Canal of the Neck of the Uterus, by Ellwood Wilson, M. D., are interesting subjects which are considered. An index of Obstetrics and Gynecological Literature of all countries for the six months preceding January 1st, 1876, is given in the volume and it is proposed to present a similar one for the year 1877, in the next volume of Transactions. All specially interested in Gynecology will much desire to read the volume.

